# The Role of Virtual Triage in Improving Clinician Experience and Satisfaction: A Narrative Review

**DOI:** 10.1089/tmr.2023.0020

**Published:** 2023-07-31

**Authors:** George A. Gellert, Joanna Rasławska-Socha, Natalia Marcjasz, Tim Price, Alicja Heyduk, Agata Mlodawska, Kacper Kuszczyński, Aleksandra Jędruch, Piotr Orzechowski

**Affiliations:** ^1^ Evidence-Based Impact and Value Demonstration, Infermedica Inc., San Antonio, Texas, USA.; ^2^ Clinical Validation and Evidence-Based Impact and Value Demonstration, Infermedica Inc., Wrocław, Poland.; ^3^ Product Development, Infermedica Inc., London, United Kingdom.; ^4^ Implementation and Customer Success, Infermedica Inc., Wrocław, Poland.

**Keywords:** clinician satisfaction, virtual triage, symptom checker, clinician experience, artificial intelligence

## Abstract

**Objective:**

This review examines the literature on improving clinician satisfaction with a focus on what has been most effective in improving experience from the perspective of clinicians, and the potential role that virtual triage (VT) technology can play in delivering positive clinician experiences that improve clinical care, and bring value to health care delivery organizations (HDOs).

**Methods:**

Review and synthesis of evidence on clinician satisfaction indicating a potential for VT to favorably impact clinician experience, sense of effectiveness, efficiency, and reduction of administrative task burden. Analysis considers how to conceptualize and the value of improving clinician experience, leading clinician dissatisfiers, and the potential role of VT in improving clinician experience/satisfaction.

**Results:**

Contributors to poor clinician experience/satisfaction where VT could have a beneficial impact include better managing resource limitations, administrative workload, lack of care coordination, information overload, and payer interactions. VT can improve clinician experience through the technology's ability to leverage real-time actionable data clinicians can use, streamlining patient–clinician communications, personalizing care delivery, optimizing care coordination, and better aligning digital/virtual services with clinical practice. From an organizational perspective, improvements in clinician experience and satisfaction derive from establishing an effective digital back door, increasing the clinical impact of and satisfaction derived from telemedicine and virtual care, and enhancing clinician centricity.

**Conclusions:**

By embracing digital transformation and implementing solutions such as VT that focus on improving patient and clinician experience, HDOs can address barriers to delivery of high-quality, efficient, and cost-effective care. VT is a digital health tool that can create a more streamlined and satisfying experience for clinicians and the patients they care for. VT is a technology solution that can help clinicians make faster more informed decisions, reduces avoidable care, improves communication with patients and within care teams, and lowers their administrative burden so they have more quality time to care for patients.

## Introduction

When clinicians have poor experiences with information technology, it combines with an increasing administrative burden and contributes to professional burnout and diminished job satisfaction, which can have unintended repercussions for patient care and outcomes.^1^ Enabling a positive work experience for clinicians is crucial but challenging, often requiring new organizational capabilities, new processes, and deployment of new technologies. Barriers such as legacy systems, outdated processes, and organizational culture can undermine attempts to refresh the health care delivery experience for clinicians.

However, health care delivery organizations (HDOs) that choose to prioritize clinician experience will be better positioned to improve clinical excellence and gain market share, while reducing clinician burnout and turnover. This review examines and integrates the literature on improving clinician satisfaction with a focus on what has been most effective in improving health care experience from the perspective of clinicians, and the potential role that virtual triage (VT) technology can play in delivering positive clinician experiences that foster clinician retention and loyalty, improve clinical care, and bring value to HDOs.

HDOs are bringing more of their services online in the form of patient portals and digital front doors where VT is implemented to serve as the first point of contact for patients (or family members) with a health care delivery system. Individuals using a smartphone, tablet, or laptop to assess their symptoms on popular browsers are connecting with essentially random internet health care content of questionable scientific and clinical accuracy. However, a systematic VT engine is typically a multispecialty physician developed and certified digital tool that enables patient-users to more confidently and accurately secure a preliminary assessment of their symptoms, and which conveys reliable, actionable clinical care guidance.

VT is a digital technology that can be engaged by patients 24/7/365 and is accessible from any device with internet connectivity to assist in evaluating their symptoms and determining the appropriate level or acuity of care that is needed. A VT classification algorithm considers the severity of the most likely conditions identified by an inference engine, as well as the occurrence of any particularly acute and alarming symptoms or risk factors.

The inference engine within VT software computes the probabilities of likely conditions and automatically selects the most relevant questions to ask the patient-user next. At the heart of a VT inference engine is a statistical algorithm for advanced symptom assessment based on Bayesian networks that use the underlying knowledge stored in a medical knowledge base. The inference algorithm identifies which question to ask based on the patient's previous answers, demographics, medical history, and risk factors, using a value of information criterion. There are no prescribed interview paths, and thus, in light of new evidence, the system can switch between various hypotheses (as human physicians do).

The primary function of a VT application is the conduct of a preliminary medical interview during which the patient-user answers questions regarding their sense of well-being and symptoms experienced. The answers provided are analyzed on a current basis, and within a few minutes, VT software conveys the analysis of symptoms and notifies the patient-user of probable causes, severity, and recommended level or acuity of medical care warranted. VT applications ask the patient-user to provide basic information, such as age, sex, risk factors, medical history, and symptoms.

On the basis of these responses, the preliminary medical interview asks questions adjusted to the patient-user's specific case, focusing on the intensity of the symptoms and their occurrence, and other related symptoms. The interview concludes with a user-friendly presentation of symptom analysis results and a recommendation for one of four levels of care, including self-care, consult a physician on an outpatient basis when next possible, proceed to an emergency department (ED), or call an ambulance for transport to an ED. A VT application is fully scalable in terms of both the number of users and the volume of medical content.

VT is superior to a generic internet browser search because patients are evaluated to identify and convey a triage level using an evidence-based set of clinical algorithms advising if—and how quickly—they should consult with a physician or other care provider, or if they could instead engage self-care. In a few minutes, patients can verify their health status and care needs leveraging information and artificial intelligence (AI) that has been extensively vetted and validated by physicians. VT enables health systems to provide patients with access to information and services beyond regular workday and daytime physician office working hours, or that involves potentially challenging travel to and possibly long waits in an ED. VT can be especially helpful when individuals are uncertain if their symptoms warrant medical/physician or nurse attention, if and when their symptoms worsen, and when they are unsure of what to do.

Patients may struggle to navigate the complexity of available medical services—which can be exacerbated given high variability in cost and health insurance coverage. Commencing a patient's health care journey with VT can help provide patients guidance on available and clinically suitable health care services, and when well integrated within the digital front door of a health care delivery system, can direct patients to very specific services and care settings available within the user's network that have been informed by the patient's clinical presentation and needs. VT thus has the potential to either accelerate obtaining needed acute care or reduce unnecessary health care seeking in settings of greater acuity, complexity, and cost than the individual's illness and chief complaint warrants from a clinical viewpoint.

While VT enables patients to more rapidly secure appropriate care, effective clinical triage can also potentially reduce patient volume pressure in high-demand service lines, such as the ED, improve the efficiency/appropriateness of clinical staffing, and possibly reduce clinician stress and dissatisfaction resulting when a large volume of patients present during already busy ED shifts that do not require emergent care. VT deployed strategically in health systems can also potentially help reduce patient leakage and maintain patients' engagement within a system network. Table 1 contrasts key differentiating features of general internet browser searches and VT in educating and guiding patient-users.

**Table 1.** Comparison of Random Internet Medical Content Searches with Artificial Intelligence-Driven Virtual Triage

Characteristic	Common internet Search engines	Evidence-driven VT
Input information	Single key words/phrases	Responses to highly specific clinical queries about multiple coexisting symptoms, pertinent medical history, and risk factors processed through algorithms engaging AI
Information produced and conveyed to patient-users	Random information, unrelated to and uninformed by the user's clinical case and information	Evidence-based health assessment based on patient-user clinical, medical history, and demographic data
SEO	Based on the most popular searches and nonclinical SEO optimization	Independent of SEO, focused on and informed by individual user case and clinical information/symptoms and scientific evidence
Quality of clinical assessment and health care referral guidance	Difficult to assess, especially without medical knowledge and clinical understanding	Confirmed by specialty board-certified and licensed physicians, as part of a clearly defined and audited evidence-based quality management system
Time required to receive actionable information and clinical guidance	Varied, depending on the quality of results produced by search and the number of topics/issues searched	Several minutes (4–7), after which specific and clinically actionable recommendations are presented
Resulting health care seeking potential actions and pathways	Unknown and not informed by a robust scientific evidence base and clinical expertise	Specific care referral to acuity-appropriate and available clinical care services
Scientific and clinical integrity of care recommendations generated	Mixed, frequently built around worst possible clinical scenarios found online (negative selection bias)	Objective and evidence-based recommendations, conveying reliable clinical guidance and safest care referral

AI, artificial intelligence; SEO, search engine optimization; VT, virtual triage.

This analysis approaches the topic of the potential of VT to improve clinician experience and satisfaction as a narrative rather than a systematic review, because initial examination of the literature indicated that the volume of published peer reviewed studies and journal literature—and particularly quantitative studies evaluating the impact of VT on clinician experience/satisfaction was slight and not substantive or extensive enough to warrant, or to enable, a systematic review of the literature.

## Methods

The primary objective of this review was to describe the use of VT in the context of clinician experience and satisfaction. Due to the small amount of available literature and studies on this particular topic, a narrative review was undertaken to provide an overview of the potential of VT to improve physician experience and satisfaction, and which may serve to identify important areas of further research.

A thorough literature search was performed using the following keywords and related terms, including MeSH terms: *virtual triage*, *symptom checker*, *digital triage*, *physician satisfaction*, *clinician satisfaction*, *artificial intelligence*, *physicians*, *clinicians*, *machine learning*, *burnout*, *healthcare workers*, *life satisfaction*, *medical informatics*, *virtual appointment*, *mobile application*, *electronic health records*, *team-based care*, *patient care*, *diagnosis*, *computer-assisted methods*, *workflow*, *communication*. Our narrative review of the literature was specifically and exclusively focused on “virtual triage” technology, also commonly referred to as “symptom checkers,” which is an automated, non-live encounter process driven by AI integral to the technology platform.

Excluded were such nonspecific, general, and inclusive search terms as “telemedicine, telehealth, eHealth,” which produce an impossibly large and nonspecific number of articles to review and assess, few of which actually focus specifically on VT and symptom checkers. Furthermore, while PubMed identifies 57 potential articles for review when the search terms “tele-triage” or “teletriage” are used, this almost uniformly selects articles focused on the use of telephonic, live agent remote clinical triage, which is completely different from the automated, AI-driven technology deployed in VT. However, we have identified and included publications related to telehealth and clinician practice to provide a broader context for the research topic. The following databases were searched: Google Scholar, PubMed, Science Direct, ResearchGate, and Google. Literature was also identified by cross-referencing from key articles.

The major challenge in completion of the literature search and review was the small number of peer reviewed journal publications focusing on clinician satisfaction in the context of using VT tools, in part reflecting that it is an innovative and novel technology. Included were articles describing the features and opportunities of VT use and its influence on clinician satisfaction and use in clinician practice. Articles were included based on the following criteria: (1) examination and discussion of important aspects of VT use contributing to the level of clinician satisfaction (e.g., professional burnout, workload, administrative tasks, communication quality, clinician self-efficacy, clinician time management); and (2) examination and discussion of important aspects of AI-based VT use in clinician practice (e.g., disease prevention, clinical diagnosis, clinical documentation, case management, clinical follow-up, clinical workflow, clinician communication, patient care).

Journal articles were sought and integrated into the review from 2014 onward. Of the 77 reference citations included, 20 were published before 2020. Articles on VT that pre-date 2020, after which there have been major advances in the AI that drives VT—describe an obsolete technology far more primitive than the technology developed and reported on in the literature from 2020 onward. Thus, our primary focus was on 2020 and later reports published that identified the potential for VT to positively impact clinician satisfaction. Sources from before 2014 were included to share background on the evolution of AI technology in medicine, and do not necessarily mention VT, but have enabled us to outline the background for the entire article.

Articles in languages other than English were excluded, as were those published before 2008. Other article exclusion criteria included articles not covering VT and related technology solutions in the context of clinician satisfaction and practice, studies focusing on aspects of technology measure implementation other than those described in the research topic, duplicate articles, and case studies.

For each article identified, the titles, abstracts, and keywords were screened for relevance. Articles selected for inclusion in the review were read in full text independently by one or more of three researchers, reviewed, discussed, and summarized. Articles were included in the final literature review and synthesis based on the consensus of the reviewers. To create as comprehensive a picture of the discussed issues as possible despite the limited literature on the subject, it was decided to include select non-peer reviewed publications and other online publications after a thorough vetting and verification of the reliability, validity, and credibility of cited sources. All articles were reviewed by at least three individuals, and final article inclusion or exclusion was decided by the lead author. All co-authors contributed to the integration of the included articles' findings into the review, as well as development and expression of the primary review theme of the potential value of VT in improving clinician experience and satisfaction.

## Results

### Conceptualizing clinician experience in the context of virtual triage

The clinician experience encompasses every facet of provider engagement during both direct and indirect clinical care delivery. It involves diverse components from medical chart review and use of the electronic health record (EHR), to complying with payer requirements and personal encounters with patients and their families. In addition, administrative tasks, follow-up telephone calls, and chart reviews continue well after patient visits/rounds have ended. Clinician experience is affected by a constantly evolving health care landscape, continuous expansion of information—with medical knowledge today doubling every 73 days, compared with every 50 years in 1950—and rapidly changing expectations of patients.^2^ Given these challenges, a focus on optimizing clinician experience by streamlining information and clinical workflows is central to a satisfied clinical workforce that provides high-quality efficient medical care.

VT can facilitate more efficient communication with patients and greater continuity of patient care, and can accurately stratify and prioritize patients in terms of clinical urgency and appropriate level of care based on acuity need.^3^ The AI within VT refers patients directly to an appropriate specialty physician, nurse, or other clinicians and identifies patients who may engage in self-care. Patient waiting lists can be shortened, enabling more efficient appointment management and reducing the burden on clinician schedules^4^ and ED operations. VT can potentially reduce avoidable hospital admissions and avoidable complications through early detection of prodromes indicating potential serious illness, reducing avoidable care utilization and costs.^5^ In addition, the stress associated with complex, unassisted clinical decisions can be reduced by using VT that supports care decision-making processes.^6,7^ By matching patients to the appropriate acuity level, health care capacity can be expanded despite staffing limitations, at lower cost than employment of new personnel.^8^

The complexities of health care delivery and high demands on clinicians greatly impact their experience, with potentially detrimental effects on patient outcomes. The American Medical Association describes physician burnout as epidemic in the United States, with 44.0% of physicians reporting signs of burnout at least weekly.^9^ Professional burnout can contribute to less patient engagement, risk of increased medical errors, higher absenteeism, and early retirement. Studies report that 34.1% of nurses experienced emotional exhaustion.^10^ The COVID-19 pandemic worsened burnout, with 3 in 10 clinicians considering leaving health care.^11^

Use of new information technologies is associated with improved clinician work–life balance and reduced symptoms of burnout.^12^ Technological solutions optimize clinician workflows and time, increase efficiency, and reduce risk of medical errors.^13,14^ VT has potential to reduce clinician frustration with redundant or inappropriate patient visits, for example, when patients are improperly referred to a specialist, or referred for health problems where self-care or a general practitioner (GP) visit will suffice. Administrative tasks not directly related to patient care are an important contributor to clinician burnout. VT can facilitate preparing medical records by automatically providing past medical history (PMH) or prescription lists.^15^ By reducing clinician administrative workload, AI-based VT allows greater focus on the medical aspects of health care and patient communication, increasing clinician sense of self-efficacy, which reduces burnout.^16^

### The value of improving clinician experience

Clinicians report increased satisfaction when a patient-centered approach to care is prioritized. Increased understanding of both patient and clinician experience is essential to creating a patient-centered approach to health care. The more ways clinicians have to support patient engagement and communication, such as using VT, the more meaningful their work experience, which impacts patient satisfaction and correlates with improved clinical outcomes, medication adherence, and reduced avoidable readmissions.^17^ AI-based VT streamlines clinician–patient communication, which can potentially increase clinician satisfaction. VT benefits physician time management by shifting time/attention from administrative tasks to patient care and communication.^18^

Clinician sense of care efficiency, and provider and patient satisfaction, are complexly interrelated. Provider and patient satisfaction are complexly interrelated. Positive patient experiences are associated with a lower risk of medical malpractice; one analysis demonstrated a 21.7% increase in being named in a malpractice suit when patient experiences were poor.^19^ Improved patient experience is associated with greater provider satisfaction and reduced turnover, with a 4.7% reduction in provider turnover reported after improvement in patient experience.^19^

Physicians report that technology use has improved patient care by allowing greater ease of access to medical chart information and reducing medication errors.^20^ Incorporating new technology into clinical workflows has also eased the administrative burden on clinicians, opportuning higher quality interaction with patients. AI-assisted VT can automatically provide care referral and preventive recommendations based on a patient's age, symptoms, comorbidities, and risk factors, conveying the clinician additional time to deepen the patient's history and to answer patient questions, thus implementing a more patient-centered approach to care.^21^ VT allows remote evaluation of treatment needs that can favorably influence the patient's active participation in the treatment process, benefiting the care relationship. VT encourages proactive patient attitudes that facilitate improved interaction with clinicians and can reduce risk of learned helplessness negatively impacting treatment outcomes.^22^

### Contributors to clinician dissatisfaction

#### Resource limitations

Clinician satisfaction is undermined by inadequate resources, causing providers to feel pulled in many directions, as they work longer hours with greater nonclinical administrative tasks, all contributing to professional fatigue and burnout. A 2020 U.S. survey of 597 physicians found that 36.0% had assumed new nonclinical roles and responsibilities during the COVID-19 pandemic.^23^ The combination of lean staffing, lack of resources and of work–life balance contributes to clinician burnout. VT can potentially improve clinician time management by reducing clinician workload and combating short consultation times that make it difficult to complete a detailed evaluation of risk factors.^24^

#### Administrative workload

Clinicians manage numerous administrative tasks that divert time and attention away from patient care. Administrative work consumes one sixth of U.S. physicians' work time and lowers provider satisfaction.^25^ From a financial viewpoint, physicians completing administrative tasks are costly and should be delegated to the nonphysician workforce or automated to enable physicians to focus on delivering patient care. The amount of administrative work required of primary care physicians has decreased student interest in health care as a potential occupation, causing a projected U.S. shortage of physicians.^26^ Nurses also report spending one third of their time on administrative tasks.^27^

Enhanced documentation tools and patient intake solutions streamline patient data collection and reduce and expedite administrative tasks that allow clinicians to focus on patient care, yielding increased patient and clinician satisfaction. The use of AI-based solutions such as VT makes it possible to automate administrative tasks in health care without need to train or hire additional personnel,^28^ supporting clinicians in documentation and referrals to other specialists.

#### Lack of care coordination

Clinicians need tools to assist in streamlining care in the complex ecosystem of health systems. Making referrals to and communication between various providers to coordinate care requires time and health system knowledge. Clinicians struggle to get the appropriate level of care for patients from the right specialty provider,^29^ negatively affecting their ability to provide efficient, timely patient routing to subspecialties, a key to improving clinical outcomes.

VT can serve as an effective tool to navigate and route patient care if used as a digital front door, including an initial interview that reduces wait times for a specialist. AI-powered VT can identify patients in need of urgent medical care and recommend calling for an ambulance or visiting an ED. Used in care coordination, VT can improve communication between care professionals to reduce unnecessary diagnostic and therapeutic procedures.^30^ VT can accelerate the diagnostic process^31^ and can collect and transmit medical data in a standardized, intuitive, and readable form that facilitates communication and coordination between clinicians, enabling safer patient transfers and interdisciplinary case management.^32^

#### Information overload

The amount of patient clinical information that is generated/updated daily is substantial and challenges clinicians' ability to provide high quality care. Use of decision support tools such as AI-based VT incorporates the latest medical updates, enabling clinician confidence in providing evidence-based, consistent, and high-quality care to patients. VT can assist physicians in difficult clinical cases, such as patients with suspected rare diseases^33,34^ or presenting nonspecific symptoms, and can assist in differential diagnosis.^35^

#### Payer interactions

Physicians must navigate insurance companies and payers, obtaining prior authorizations for medications and procedures and addressing denials, among other tasks. These activities divert clinicians from patient care and consume time, with physicians spending an average of 3.5 h per week interacting with payers, at an estimated cost of $31 billion annually in the United States.^36^ VT can automatically check the type of health insurance plan, verify covered medical procedures, and send automated queries or required patient information to payers.^37^

### Improving clinician experience and satisfaction: technology as part of the solution

Solutions to reduce clinician administrative workload must be rapidly deployable, simple to configure and customize, easy and intuitive to learn, and proven in clinical environments.^38^ VT meets these requirements. The COVID-19 pandemic fostered widespread adoption of telehealth and remote patient monitoring, with providers rapidly scaling telehealth offerings and seeing 50–175 times the number of remote patients than pre-pandemic.^39^ Patients welcomed this new accessibility and convenience, with 88% wanting to continue using telehealth for nonurgent consultations as COVID-19 receded,^40^ benefiting providers as well. VT allows for precise targeting of the medical interview by integrating additional, often complex data, directing the patient to the right level of care, the right specialist, and the right form of consultation.^41^

During the COVID-19 pandemic, clinicians appreciated the flexibility of conducting virtual patient care and monitoring, including ability to improve scheduling and prioritization of appointments.^12^ Use of VT reduces clinician exposure to infectious agents by remotely managing patients' health problems.^42^ This was particularly important during the pandemic in reducing absenteeism among medical personnel and maintaining productivity.^43^ VT enables continuity of care in facilities, such as nursing homes and prisons,^44^ and can reduce the number of in-person home visits needed for patients who are immobile, disabled, bedridden, or living in rural areas.^45^ The pandemic clearly demonstrated that a large proportion of care can be delivered virtually, caring for patients where they are, with greater convenience and less effort.

VT can help HDOs improve the care experience for clinicians through the valuable functionality summarized in Table 2.

**Table 2.** Value of Virtual Triage Functionality in Improving Clinician Experience and Satisfaction

1. Leverages real-time actionable data clinicians can use
2. Streamlines patient–clinician communication
3. Personalizes care delivery
4. Optimizes care coordination
5. Aligns digital/virtual services with clinical practice
6. Contributes to a digital back door
7. Increases impact of and satisfaction derived from telemedicine and virtual care investments
8. Helps create and enhance clinician centricity

#### Leveraging real-time actionable data clinicians can use

Providing patient data *per se* is no longer sufficient in efforts to improve clinician experience and satisfaction—it needs to be collected in a way that is intuitive, secure, and actionable. Data capture by VT analyzes the patient's clinical presentation and PMH and presents the findings ranked in order of probability, helping clinicians more rapidly and cost-effectively digest patient information in their care management. VT should be an integral component of HDO efforts to systematically capture, organize, and constructively leverage real-world actionable patient data in real time.

#### Streamlining patient–clinician communication

Given the importance of communication in health care, solutions that facilitate better interactions between patient and provider can add considerable value, whereas legacy systems, siloed data, lack of integration, and misdirected or ignored messages cause communication gaps, duplications, and breakdown. With VT, providers can streamline communication, automate and personalize key parts of the patient journey such as care referral, and configure communications to better meet patient preferences and literacy levels. VT streamlines communications and facilitates faster, better care delivery and a more satisfying experience for provider and patient. VT can help collect, process, and present the most immediately relevant medical data to clinicians,^46^ allowing them to focus on the clinical essence of a patient's health concern, positively impacting clinician confidence and contributing to clinician satisfaction.

#### Personalizing care delivery

The increased availability of social, environmental, and behavioral determinants of health data, and the application of machine learning to these data sets, are having a major impact on how care services can be personalized to the specific needs of individual patients. From medications and dietary considerations to daily living and personal communication preferences, providers can now build a holistic picture of patients and proactively recommend appropriate therapeutics, services, and education that align with where they are in their health care journey. Key to improving both the clinician's and patient experience through a patient-centered approach is implementing technology solutions that relieve clinicians of administrative tasks.^47,48^

VT can, based on identified medical conditions and risk factors, autonomously create and customize patient care guidance and education such as need for screenings, vaccinations, or prescription renewal.^49,50^ AI-based VT can tailor preventive recommendations for patients based on their clinical data, demographics, risk factors, and health profile, and engage education to improve patient lifestyle, exercise level, or diet.^51^ VT can capture the most urgent and life-threatening data, including cardiovascular events,^52^ violence, suicide risk,^53,54^ or premature birth,^55^ and initiate a rapid emergency system response. VT automates prescriptions and care recommendations and notifies patients of special health issues, such as history of anaphylaxis or medication expiry date,^56,57^ simplifying/expediting clinician workflows and thereby increasing professional satisfaction.

#### Optimizing care coordination

Suboptimal care coordination impacts many aspects of clinician experience and satisfaction, can lead to poor care outcomes, and hinders HDO efficiency and revenue. Lack of coordinated care may contribute to higher patient morbidity, avoidable hospital admissions, and acute readmissions that are costly to patients, payers, and health systems.^58^

VT can eliminate barriers and bottlenecks in care delivery by automating communication, removing redundancies in information capture/exchange, and improving patient flow. Given the need to improve the quality of communication between different levels of care, and between GPs and specialists,^59^ VT expedites seamless, high quality, and focused communication among clinicians, provides automated feedback, such as from a specialist to a GP, from a physician to a nurse, or among specialists—all clinician satisfiers. Improved access to patient medical data through VT can also support detection and care referral of domestic violence and child abuse,^60^ Munchausen syndrome,^61^ and substance abuse.^62^

#### Aligning digital/virtual services with clinical practice

To be clinician satisfiers, digital tools need to align with clinical workflows and be simple to understand and use. VT integrates well into existing organizational processes, revenue cycle management, and clinical workflows. Poor integration risks disengaging clinicians, as evidenced by a KLAS survey finding that clinicians very dissatisfied with their HDO EHR are three times as likely to leave the health system compared with those who are satisfied.^63^ Delivering a consistent experience across asynchronous and synchronous service components and patient information sources is key to offering a great clinician experience. VT supports intuitive and automated preparation of patient information, providing clinicians with information about pre-existing conditions, risk factors, and medical history in a rapid easily accessible manner. VT also offers clinicians references content on current clinical guidelines.

#### Adding a digital back door

Digital transformation in care delivery also must focus on what happens following a health care encounter. Timely post-visit follow-up and patient education deliver a high-quality end-to-end experience for both patient and provider and can drive patient adherence to treatment plans, reduce avoidable readmissions, and improve outcomes.^64^ Care follow-up solutions offer seamless automated ways for patients and providers to stay connected after a visit, helping patients secure the care guidance and support they need from providers to adhere to their treatment plan. As health care continues to migrate away from the hospital and clinic and into the home and workplace, technology in the form of virtual care services, remote patient monitoring tools, and digital engagement applications can help HDOs scale those services and deliver more personalized care—wherever and whenever the patient needs it.

Clinicians can use VT as an outpatient follow-up tool,^65,66^ enabling patient adherence monitoring,^66^ for example, in chronic diseases such as diabetes, cardiovascular disease, or cancer.^67^ VT can verify the validity of care recommendations and assess treatment progress. AI-based VT can detect complications, evaluate effectiveness of pharmacotherapy, and rapidly identify drug interactions.^68^ In pediatric care, VT supports monitoring child development, tracks achievement of developmental milestones,^69^ and assesses growth.^70^ VT enables symptomatic monitoring of postoperative patients after hospital discharge.^71^ A robust digital back door can improve clinician care experience and satisfaction.

#### A needed synthesis of patient and clinician satisfaction that is achievable through telemedicine and virtual care

Recognition of the inseparability or indivisibility of patient and clinician experience and satisfaction can be made actionable through a well integrated deployment of virtual triage, care referral and delivery capabilities and solutions. More satisfied clinicians contributes to more satisfied patients, and vice versa. Virtual technologies leverage and translate this aspiration into reality. A survey of 1.3 million patients showed that 89% were satisfied with their physician after a telehealth visit and 76% would recommend a virtual appointment after experiencing one.^72^ Despite increasing patient demand for telemedicine, it accounts for less than one quarter of patient care, suggesting that while both clinicians and patients enjoy the convenience of telehealth, logistical issues continue to impede broader adoption. Reengineering triage processes, offering VT and telehealth as options, and automating steps in the patient and clinician journey makes for a more satisfying experience for patient and clinician alike.

For common conditions (e.g., upper respiratory infections, influenza, allergies, depression and anxiety), VT and telehealth can play a substantial role,^73^ allowing clinicians to diagnose patients and issue e-prescriptions more rapidly (if needed). VTs ability to help clinicians make informed decisions rapidly and divert patients to clinically appropriate, more convenient virtual care options, rather than an in-person office appointment or ED visit can improve clinician sense of efficacy. Enabling more clinically and cost-effective use of clinician time and allowing clinicians to focus on higher acuity cases fosters clinician satisfaction.

Adopting VT requires a reframing of the way HDOs regard the care continuum, where a hybrid model of in-person and telehealth visits becomes commonplace. Among patients who regularly receive telemedical care, 80% report a virtual care experience that was as satisfying as an in-person one.^74^ A robust care management platform that supports VT and telehealth services is essential in creating a seamless and satisfying telehealth experience for patients and clinicians. With VT and telehealth care diverting lower acuity cases to virtual care when clinically appropriate, a hybrid care model reduces clinician administrative burden and streamlines their workflows, positively impacting clinician satisfaction.

#### Creating clinician centricity

While health systems struggle to implement patient- and clinician-centric solutions, clinicians have many care setting choices, with major retailers such as Walmart, CVS, and Amazon offering consumer-centric care options.^75^ HDOs need to engage the same kind of customer focus these traditionally consumer-centric competitors offer, for both patients and clinicians. For every patient visit, clinicians spend 16 min on administrative tasks including the EHR,^76^ where VT, including patient intake that streamlines patient data collection and fits seamlessly into clinical workflows, benefits both patients and providers. VT frees physicians from asking repetitive questions by collecting essential patient data before consultation, minimizing administrative tasks during the visit, and freeing more time for physicians to spend with patients.

Traditional call centers can deliver significant value, but historically have been largely a solution for managing appointment scheduling and reminders, very basic triage, and health care referrals, and have fallen short in optimizing and streamlining care delivery. Antiquated hospital-based call centers rely on costly clinical staff to provide 24/7 coverage. AI-driven VT of patient inquiries and acuity-appropriate care referral automates triage and helps optimize call center performance by diverting administrative tasks such as receiving and responding to patient calls, emails, and other communications from the care delivery team, allowing providers more time to focus on care. Leveraging VT to reduce complexity and create a digital front door to health systems delivers a more satisfying experience designed around the needs of clinicians. Integration of VT and call center operations facilitates better matching of clinical presentation with needed care acuity, including telemedicine visits or chat-based virtual consultations.

## Discussion and Future Research Directions

The primary limitation encountered in the conduct of this narrative literature review was the paucity of studies reporting quantitative impact evaluation data on the specific demonstrated value that VT had on a specific community of clinicians. A larger number of studies reported in the literature will also enable future reviews to be more comprehensive and to offer deeper insights into the potential impact and value of VT implementation in specific health care delivery settings. Overall improvements in clinician experience and satisfaction will be related to the efficacy of VT in driving more acuity-appropriate care to various health care settings, including diversion of ED use by patients with nonemergent clinical presentations that are better managed through office-based, virtual or self-care. Future research must focus on evaluating the alignment of VT condition identification and care referral with validated final diagnosis by a clinician.

Nonetheless, it is already clear that VT can serve as a decision-support tool for clinicians, in part by equipping them with initial patient data ahead of vis- its and ensuring up-to-date patient information in EHR systems. VT integrated with patient intake provides patients with pertinent information and can connect them to relevant medical services, enabling clinicians to deliver more appropriate and thus better care, and thereby improving clinician satisfaction.

Diagnostikare provides business to business and business to consumer healthcare services that enable organizations to deliver safe and high quality digital health care services, and serves as a illustration of how VT can be deployed in an innovative, successful manner that improves both clinician and patient experience. Patients unsure if they should consult with a physician were asked to complete VT. Virtual triage matched the physicians' final diagnoses in 85% of cases, and helped shorten visit time from 20 to 12 minutes.^77^ Only 30% of users subsequently scheduled an in-person visit, reducing unnecessary visits, creating more treatment capacity and expediting care of other patients. Diagnostikare used VT to achieve a 39% increase in operational efficiency, measured as the mean time from patient intake to case completion, by leveraging VT acuity level mapping and matching. This also enabled patients with urgent clinical presentations to be engaged by clinicians in just 15 min, and those with mild symptoms within 1–3 h.^77^ All of these outcomes in turn improved both clinician and patient satisfaction with services.

## Conclusions

In an era where clinicians must manage greater care complexity and nonclinical demands on their time, they need and welcome technology solutions that help them make faster, more informed decisions, reduce avoidable care, which provide transparent communication with patients and within care teams, and which lower their administrative burden so they have more quality time to care for patients. By embracing digital transformation and implementing solutions such as VT that focus on improving both the patient and clinician experiences, HDOs can address key barriers to the delivery of high-quality, efficient, and cost-effective care. AI-driven automated VT, intake, acuity-matched care referral, and follow-up are digital health tools that can be leveraged by HDOs to create a more streamlined and satisfying experience for clinicians, as well as the patients they care for.

## Abbreviations Used

AI: artificial intelligence
ED: emergency department
EHR: electronic health record
GPs: general practitioners
HDOs: health care delivery organizations
PMH: past medical history
SEO: search engine optimization
VT: virtual triage
